# Heterologous Hsp90 promotes phenotypic diversity through network evolution

**DOI:** 10.1371/journal.pbio.2006450

**Published:** 2018-11-15

**Authors:** Tracy Chih-Ting Koubkova-Yu, Jung-Chi Chao, Jun-Yi Leu

**Affiliations:** 1 Molecular and Biological Agricultural Sciences Program, Taiwan International Graduate Program, National Chung-Hsing University and Academia Sinica, Taipei, Taiwan; 2 Institute of Molecular Biology, Academia Sinica, Taipei, Taiwan; 3 Graduate Institute of Biotechnology, National Chung-Hsing University, Taichung, Taiwan; 4 Biotechnology Center, National Chung-Hsing University, Taichung, Taiwan; Massachusetts Institute of Technology, United States of America

## Abstract

Biological processes in living cells are often carried out by gene networks in which signals and reactions are integrated through network hubs. Despite their functional importance, it remains unclear to what extent network hubs are evolvable and how alterations impact long-term evolution. We investigated these issues using heat shock protein 90 (Hsp90), a central hub of proteostasis networks. When native Hsp90 in *Saccharomyces cerevisiae* cells was replaced by the ortholog from hypersaline-tolerant *Yarrowia lipolytica* that diverged from *S*. *cerevisiae* about 270 million years ago, the cells exhibited improved growth in hypersaline environments but compromised growth in others, indicating functional divergence in Hsp90 between the two yeasts. Laboratory evolution shows that evolved *Y*. *lipolytica*-*HSP90*–carrying *S*. *cerevisiae* cells exhibit a wider range of phenotypic variation than cells carrying native Hsp90. Identified beneficial mutations are involved in multiple pathways and are often pleiotropic. Our results show that cells adapt to a heterologous Hsp90 by modifying different subnetworks, facilitating the evolution of phenotypic diversity inaccessible to wild-type cells.

## Author summary

Biological processes in living cells are often carried out by gene networks. Hubs are highly connected network components important for integrating signal inputs and generating responsive functional outputs. Heat shock protein 90 (Hsp90), a versatile hub in the protein homeostasis network, is a molecular chaperone essential for cell viability in all tested eukaryotic cells. In yeast, about a quarter of the expressed proteins are profoundly influenced when Hsp90 activity is reduced. Despite its pivotal role, we found that the function of Hsp90 has diverged between two yeast species, *Yarrowia lipolytica* and *Saccharomyces cerevisiae*, which split about 270 million years ago. To understand the impacts and adaptive strategies in cells with an altered network hub, we conducted laboratory evolution experiments using a *S*. *cerevisiae* strain in which native Hsp90 is replaced by its counterpart in *Y*. *lipolytica*. We observed different fitness gain or loss under various stress conditions in individual evolved clones, suggesting that cells adapted via different evolutionary paths. Genome sequencing and mutation reconstitution experiments show that beneficial mutations occurred in multiple Hsp90-related pathways that interact with each other. Our results show that a perturbed network allows cells to evolve a broader range of phenotypic diversity unavailable to wild-type cells.

## Introduction

Biological functions are often considered the collective output of genes interacting coordinately in a network [1–4]. Although most genes in a network only have a few links, some “hub” genes are highly connected to network components and play a vital role in maintaining intra-network connectivity [2]. As a consequence, hub genes usually possess pleiotropic functions and have a profound influence on cell fitness [5, 6]. Phenotypic variation was enhanced in yeast cells in which hub genes were disrupted, revealing a role for hub genes in network robustness against genetic or environmental perturbations [7]. Comparative genomics has shown that some hub genes can diverge significantly at the sequence level. However, little is known about to what extent the function of hub genes can change over long-term evolution or how changes in hub genes influence the evolutionary trajectory of an entire network.

The molecular chaperone heat shock protein 90 (Hsp90) represents a well-documented network hub. Hsp90 is present in bacteria, fungi, plants, and animals [8] and is essential for cell viability in all studied eukaryotic species [9–13]. Large-scale studies in *S*. *cerevisiae* indicate that Hsp90 is one of the central hubs in both physical and genetic interaction networks [14, 15]. More than 10% of the whole yeast and human proteomes have been shown to be clients of Hsp90 [16]. Moreover, the yeast Hsp90-dependent proteome is enriched with other hubs and essential genes [17].

In general, Hsp90 acts at a late stage of protein folding. It assists partially folded clients to reach structural maturation [16], or it stabilizes the components of multi-subunit protein complexes and facilitates their assembly [18–20]. Hsp90 has a high degree of specificity for client recognition, which is attributed to its sophisticated co-chaperone system [21, 22]. Interestingly, it has been found that the activity or stability of some Hsp90 client proteins can become Hsp90 independent after introducing mutations [23, 24], suggesting that the link between Hsp90 and its clients may be changed over time.

Apart from these physiological functions, Hsp90 has been suggested to buffer genetic and nongenetic variations to ensure developmental stability in fungi, plants, and animals [25–28]. Organisms exhibit constant phenotypes under normal conditions, even if they host genetic or nongenetic variations. However, when Hsp90 activity is compromised or titrated by extreme environmental stresses, heterogeneous phenotypes are revealed in a population. This increased phenotypic diversity is hypothesized to help the population survive or adapt during drastic environmental changes [29].

To understand the implications of hub evolution, we replaced the Hsp90-coding gene of *S*. *cerevisiae* with orthologs from other yeast species and examined their functions. *S*. *cerevisiae* cells carrying *Y*. *lipolytica-*Hsp90 (Ylip-Hsp90) exhibited decreased fitness in most tested conditions but improved growth in high-salt medium, suggesting that the function of Ylip-Hsp90 has differentiated from its *S*. *cerevisiae* ortholog. We further evolved the Ylip-Hsp90–hosting strain to study how cells respond to a changed network hub. The evolved clones adopted various evolutionary trajectories, but none involved changing the heterologous Hsp90 per se. Furthermore, fitness measurements in different stress environments showed that these clones had evolved diverged phenotypes that differed from each other and also deviated from wild-type *S*. *cerevisiae* cells.

## Results

### Replacing an essential hub gene *HSP90* with the *Y*. *lipolytica* ortholog increases salt tolerance of *S*. *cerevisiae* cells

**Fig 1:**
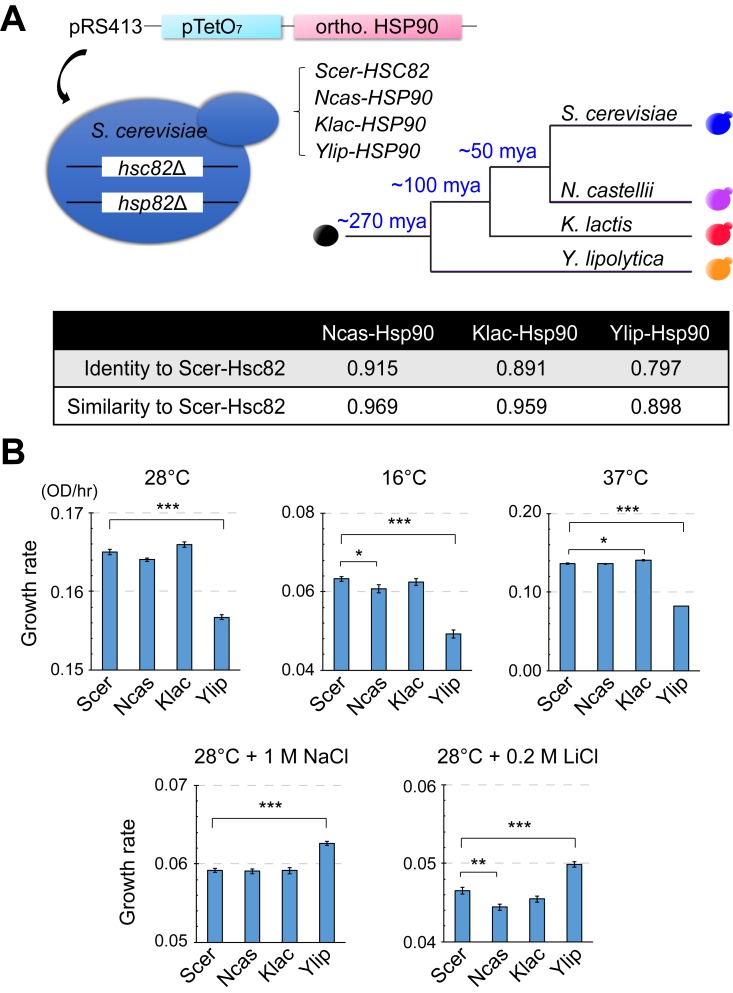
Orthologous gene replacements reveal functional divergence of Hsp90 among different yeast species. (A) Construction scheme of the *HSP90* ortholog replacement strains and phylogenetic information on the ortholog-providing species. Both copies of Hsp90-coding genes in *Saccharomyces cerevisiae* were deleted and the Hsp90 function is performed by the orthologs from other species. The bottom table shows pairwise amino acid sequence identity and similarity between Scer-Hsc82 and its orthologs (BLOSUM62, EMBOSS Water local alignment). (B) *Ylip-HSP90* replacement strains show reduced fitness in YPD media at normal, low, or high temperature but increased fitness in the YPD media containing NaCl or LiCl at 28°C. Cells were grown in liquid cultures and growth rates were measured by Infinite 200 plate readers. *p*-values were calculated by two-tailed Student *t* test with the Benjamini-Hochberg correction. *p < 0.05, **p < 0.01, ***p < 0.001. Error bar represents the standard error, *N* ≥ 4. The numerical data used in panel (B) are included in S1 Data. See also S1 Fig. BLOSUM62, BLOcks SUbstitution Matrix 62; EMBOSS, European Molecular Biology Open Software Suite; Klac, *Kluyveromyces lactis*; mya, million years ago; Ncas, *Naumovozyma castellii*; OD, optical density; ortho., ortholog; pRS413, yeast single-copy plasmid; pTetO7, tetracycline operator 7 repeats promoter; Scer, *Saccharomyces cerevisiae*; *Ylip*, *Yarrowia lipolytica*; YPD, Yeast extract-Peptone-Dextrose.

Hsp90 is an essential hub protein that interacts with 10%–20% of the whole yeast proteome, and changes to Hsp90 are probably constrained by the function of its client proteins or even the network structure [16]. To investigate whether Hsp90 protein function has changed during evolution, we replaced the endogenous Hsp90 gene in *S*. *cerevisiae* with the plasmids carrying its orthologs from *Naumovozyma castellii* (Ncas), *Kluyveromyces lactis* (Klac), and *Y*. *lipolytica* (Ylip), which diverged from the common ancestor of *S*. *cerevisiae* (Scer) at different times [30, 31] (Fig 1A). Our previous data showed that the original promoters of the orthologs from distant species often do not function properly in *S*. *cerevisiae* cells. Therefore, we placed the coding sequences of *Scer-HSC82* (one of the genes encoding Hsp90 in *S*. *cerevisiae*) and its orthologous genes under a strong tetracycline operator 7 repeats (*TetO*_*7*_) promoter, which enabled us to study changes in protein function and to also control expression levels. We then examined these Hsp90-ortholog replacement strains (denominated as *Ncas-HSP90*, *Klac-HSP90*, and *Ylip-HSP90*) under different growth conditions.

To examine how orthologous Hsp90 proteins influence the physiology of hosting *S*. *cerevisiae* strains, we measured the fitness of replacement strains under 17 different growth conditions using spot assays (see Materials and methods). For most test conditions, Ncas-Hsp90 and Klac-Hsp90 hosting strains exhibited similar fitness as Scer-Hsc82–carrying cells (Fig 1, S1 Fig and S1 Table), indicating that the function of Hsp90 is not drastically different among *N*. *castellii*, *K*. *lactis*, and *S*. *cerevisiae*. In contrast, the Ylip-Hsp90 hosting strain exhibited lower fitness than Scer-Hsc82 cells under most conditions; similar fitness to Scer-Hsc82 cells in media containing 150 mM MgCl_2_, 1.8 M sorbitol, or 0.5 M NaCl; and higher fitness under two hypersaline conditions (media containing 1 M NaCl or 0.2 M LiCl, see Fig 1B, S1 Fig and S1 Table). The phenotypic differences between Ylip-Hsp90– and Scer-Hsc82–carrying cells might be due to differences in the expression level, activity, or/and specificity of these two orthologs. No significant difference was observed when the protein levels of Ylip-Hsp90 and Scer-Hsc82 were measured using western blots (S2A Fig). To test whether the activity of Ylip-Hsp90 is different than that of Scer-Hsc82, we performed a v-src kinase assay. Hsp90 is required for the activation of v-src and, therefore, by measuring the level of v-src–dependent tyrosine phosphorylation, the activity of Hsp90 can be estimated [32]. In the kinase assay, we observed similar levels of v-src–dependent tyrosine phosphorylation in Ylip-Hsp90– and Scer-Hsc82–carrying cells (S2B Fig), suggesting that the main difference between Ylip-Hsp90 and Scer-Hsc82 is the interaction specificity instead of the activity.

*Y*. *lipolytica* cells are often isolated from hypersaline environments and exhibit high tolerance to NaCl and LiCl (S2C Fig) [33, 34]. Previous research in *S*. *cerevisiae* suggested that Hsp90 plays a crucial role in salt stress tolerance by regulating the activity of its client, calcineurin [35]. Hsp90 is essential for activation of calcineurin. However, overexpression of Hsp90 also led to salt hypersensitivity because calcineurin was sequestered by Hsp90. The increased salt resistance in Ylip-Hsp90 hosting cells suggests that the interaction between Ylip-Hsp90 and calcineurin may have been changed or Ylip-Hsp90 can facilitate the activity of other proteins involved in hypersaline growth. The altered growth pattern of Ylip-Hsp90 hosting cells reveals that the function of Hsp90 has significantly diverged between *Y*. *lipolytica* and *S*. *cerevisiae*. Moreover, our findings indicate that even a central network hub like Hsp90 can change significantly over the course of long-term evolution.

### Adaptation to a heterologous Hsp90 in laboratory evolution experiments

**Fig 2:**
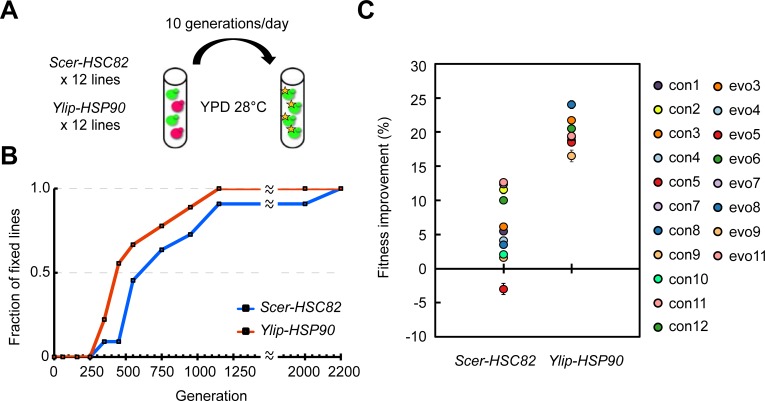
Evolving *Ylip-HSP90* lines fix the first beneficial mutations more quickly and have better fitness improvements. (A) Experimental evolution for adaptation to a heterologous Hsp90. Twelve *Ylip-HSP90* evolving lines and 12 *Scer-HSC82* control lines were set up to evolve in rich media at 28°C. Two isogenic subpopulations (red: DsRed-labeled subpopulation; green: GFP-labeled subpopulation) were mixed in an approximate ratio of 1:1 in the initial cultures. The subpopulation that obtained a beneficial mutation (noted by a star) would start expanding its frequency in the evolved culture. (B) The *Ylip-HSP90* lines become fixed more quickly than the *Scer-HSC82* lines. The mutation fixation of each culture was determined as the time when the frequency of a subpopulation was observed to be greater than 95% according to flow cytometry. The control line con6 and the evolving lines, evo1, evo2, and evo12 were excluded because of culture contamination during evolution. The frequency dynamics of each evolved line are shown in S3 Fig. (C) Evolved *Ylip-HSP90* populations have enhanced fitness improvements compared with evolved *Scer-HSC82* populations in YPD at 28°C (*p*-value = 4.54 × 10^−7^, *t* = −7.97, df = 15, one-tailed Student *t* test, unequal variance; data did not deviate from the normal distribution, Shapiro-Wilk test). Cells were grown in liquid cultures and growth rates were measured by plate readers. Fitness improvement was calculated according to the formula [(fitness of the evolved culture/fitness of the ancestral strain) − 1] × 100%. Error bars are standard errors, *N* ≥ 4. The numerical data used in panels (B, C) are included in S1 Data. DsRed, red fluorescent protein; GFP, green fluorescent protein; *Scer*, *Saccharomyces cerevisiae*; *Ylip*, *Yarrowia lipolytica*; YPD, Yeast extract-Peptone-Dextrose.

Alteration of a network hub often has a strong impact on cell fitness [5, 6]. Cells may be selected to quickly restore the original functions of the hub, or the network may be rewired to accommodate or compensate for the altered hub. In the latter scenario, it may also open the possibility for cells to evolve novel phenotypes. To explore the possible evolutionary trajectories of an altered Hsp90 network, we evolved 12 independent haploid lines carrying *Ylip-HSP90* and 12 control lines carrying *Scer-HSC82* that were all derived from the same parental *S*. *cerevisiae* clone. These cell lines were grown in regular rich medium (Yeast extract-Peptone-Dextrose [YPD]) at 28°C so that their major selective pressure is to restore the general growth defects caused by *Ylip-HSP90*. In each evolving culture, two isogenic strains carrying either the green or red fluorescent protein marker (Fig 2A) were mixed at an approximate initial ratio of 1:1. By monitoring the proportions of the two fluorescence-labeled populations at different time points, we could estimate when the first beneficial mutation was fixed in each culture (S3 Fig). In the *Ylip-HSP90* lines, more than half of the cultures fixed the first beneficial mutation within 450 generations (Fig 2B). It took another 200 generations for the *Scer-HSC82* lines to reach similar proportions of fixation. This outcome is consistent with the assumption that *Ylip-HSP90* hosting cells were initially under high selective pressure to resolve the incompatibility between the heterologous Ylip-Hsp90 and its interactive network in the *S*. *cerevisiae* background. In contrast, the *Scer-HSC82* lines only experienced very low or no selective pressure, because the evolution experiments were conducted under a normal growth condition.

After evolving for around 2,200 generations, three *Ylip-HSP90* (evo1, evo2, and evo12) lines and one *Scer-HSC82* (con6) line were lost because of contamination. We further analyzed the remaining evolved cultures. The fitness of evolved *Ylip-HSP90* populations in YPD at 28°C improved by 16%–24% compared with their ancestor (Fig 2C). The degree of improvement was significantly higher than that observed in the evolved *Scer-HSC82* populations, suggesting that the adaptive phenotype is likely to be *Ylip-HSP90* specific. When the fitness of evolved *Scer-HSC82* clones was compared with their ancestor, no significant difference was observed in four evolved lines (S2 Table), suggesting that both genetic drift and beneficial mutations contributed to the evolved phenotypes of *Scer-HSC82* populations.

To characterize the evolved phenotype, we isolated three single colonies from each evolved culture and measured their fitness. The colonies with the highest fitness from each population were chosen to represent the lineage for further examination (denominated E3 to E11 for evolved *Ylip-HSP90* clones and C1 to C12 for evolved *Scer-HSC82* clones). It was previously reported that *S*. *cerevisiae* cells often converge to a diploid state during laboratory evolution [36]. We examined the genome content of evolved clones using flow cytometry and found that all of the evolved *Scer-HSC82* clones had become diploid or triploid (S4A Fig). In contrast, most of the evolved *Ylip-HSP90* clones remained haploid, except for E10, which had become diploid. To ensure that the ploidy expansion observed in individual clones is representative of the whole population, we also examined the evolved cultures using flow cytometry and observed the same results. Because all the cultures were evolved under the same selective regime, the difference in ploidy observed in evolved cultures further implicates functional divergence between Ylip-Hsp90 and Scer-Hsc82. To further test the effect of Hsp90 on ploidy expansion or maintenance, we generated diploid strains by mating haploid cells and compared their fitness with haploid strains. Haploid *Ylip-HSP90*–carrying cells exhibited higher fitness than diploid *Ylip-HSP90*–carrying cells (S4B Fig). This result provides the possible reason why most *Ylip-HSP90* lines remained haploid after evolution.

### Fitness improvement in the evolved clones is specific to Ylip-Hsp90

**Fig 3:**
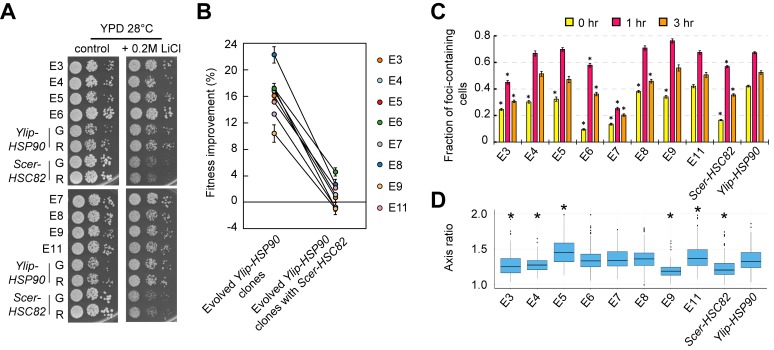
Phenotypic adaptation and innovation of the evolved *Ylip-HSP90* clones. (A) Evolved *Ylip-HSP90* clones remain highly tolerant to LiCl. Serially diluted cell cultures were spotted onto the plates and incubated at 28°C until colonies became visible. G and R indicate the ancestral strains carrying the green and red fluorescent proteins, respectively. (B) The fitness improvements of evolved clones are specific to Ylip-Hsp90. The fitness improvements were significantly diminished when the *Ylip-HSP90* gene in the evolved clones was replaced with *Scer-HSC82* (*p*-value = 6.82 × 10^−7^, *t* = 15.07, and df = 7, one-tailed paired Student *t* test; data did not deviate from the normal distribution, Shapiro-Wilk test). Cells were grown in liquid cultures and growth rates were measured by plate readers. Fitness improvement was calculated by comparing the fitness in YPD at 28°C between evolved and ancestral clones carrying the same plasmid. Error bars are standard errors, *N* ≥ 4. (C) Evolved *Ylip-HSP90* clones show different levels of protein homeostasis restoration. Cells carrying Hsp104-BFP were grown at 25°C and then shifted to 37°C to induce heat adaptation. The fraction of cells containing Hsp104-BFP foci was counted at 0, 1, and 3 hours after the temperature shift. At least seven replicates were measured for each strain, and more than 75 cells were counted for each replicate. Asterisks indicate that the fraction of cells containing Hsp104-BFP foci is significantly lower compared with ancestral *Ylip-HSP90* cells from the same time point (*p*-value < 0.01 after the Benjamini-Hochberg correction, *N* ≥ 7, one-tailed Wilcoxon rank-sum test). (D) Evolved *Ylip-HSP90* clones E3, E4, and E9 recover from elongated cell morphology, whereas E5 and E11 become even more elongated than the ancestor. The ratio of the long- versus short-axis diameter of each cell was measured, and more than 80 cells were examined for each strain. The distribution of each sample was compared to ancestral *Ylip-HSP90* cells and asterisks indicate a *p*-value <0.01 after the Benjamini-Hochberg correction by two-tailed Wilcoxon rank-sum test. The numerical data used in panels (B, C, D) are included in S1 Data. See also S5 Fig. BFP, blue fluorescent protein; *Scer*, *Saccharomyces cerevisiae*; *Ylip*, *Yarrowia lipolytica*; YPD, Yeast extract-Peptone-Dextrose.

Because the evolved clones restored the fitness of *Ylip-HSP90* cells in rich media, we wondered whether they would become sensitive to hypersaline stress like *Scer-HSC82* cells. When evolved *Ylip-HSP90* clones were tested on 0.2 M LiCl-containing plates, they displayed similar or higher fitness compared with ancestral *Ylip-HSP90* strains (Fig 3A), indicating that the *Y*. *lipolytica*-specific hypersaline-resistant phenotype had been stably maintained in the Hsp90 network of the evolved cells.

The function of Ylip-Hsp90 might be enhanced if its expression level is increased or the protein is mutated in the evolved clones. We measured protein levels of *Ylip-HSP90* using western blots. All evolved clones presented similar levels of the Hsp90 protein compared to the ancestral *Ylip-HSP90* strains (S4C Fig). We also sequenced the *Ylip-HSP90* gene of all evolved clones and found no mutation in any of them. Together, these data indicate that the evolved cells had not improved their fitness by modifying the *Ylip-HSP90* gene.

The observed fitness improvement in evolved *Ylip-HSP90* clones might be specific to the heterologous Hsp90 or simply be due to mutations that enhance general cell proliferation (e.g., loss of the costly genes or gain of glucose uptake genes). To differentiate between these two possibilities, we replaced the *Ylip-HSP90* gene of the evolved and ancestral clones with *Scer-HSC82* and measured their fitness in YPD at 28°C. The extent of fitness improvements was drastically diminished when Ylip-Hsp90 was replaced (Fig 3B and S2 Table), indicating that the beneficial effects of evolved mutations are dependent on Ylip-Hsp90.

### Protein homeostasis improves to varying degrees in individual evolved *Ylip-HSP90* clones

Because Hsp90 is an important molecular chaperone involved in maintaining protein homeostasis, we suspected that the reduced fitness of ancestral *Ylip-HSP90* cells in normal conditions might reflect disturbed internal proteostasis caused by the compromised Hsp90 network. We used an Hsp104 aggregation assay to investigate this possibility [37]. Hsp104 is a disaggregase that localizes to protein aggregates upon heat stress and helps with the disaggregation and refolding process. Ancestral and evolved cells containing fluorescent Hsp104-BFP fusion protein were first grown at 25°C and then shifted to 37°C for high-temperature adaptation. By monitoring the fraction of cells containing Hsp104-BFP foci during heat treatment, we could assess how well protein homeostasis is maintained.

Ancestral *Ylip-HSP90* cells contained more Hsp104-BFP foci than *Scer-HSC82* cells before and during heat treatment, suggesting that *Ylip-HSP90* cells indeed have a high endogenous proteome burden even before heat treatment (Fig 3C and S5A Fig). Interestingly, even though all evolved *Ylip-HSP90* clones had improved their fitness to the wild-type level, not all of them could restore their ability to efficiently clear protein aggregates. E11 cells exhibited a disaggregation pattern similar to ancestral *Ylip-HSP90* cells. In contrast, E7 cells had lower levels of Hsp104-BFP foci at initial and later time points compared with *Scer-HSC82* cells (Fig 3C, S5A and S5B Fig). Our results suggest that different evolved lines probably took different paths to fix the compromised Hsp90 network.

In addition to attenuated protein homeostasis, ancestral *Ylip-HSP90* cells also exhibited an elongated cell morphology that has previously been observed in cells lacking sufficient Hsp90 activity to stabilize its client proteins [28]. When we examined the cell morphology of evolved *Ylip-HSP90* clones, we found that only E3, E4, and E9 cells recovered from the elongated cell morphology. E5 and E11 evolved to an even more elongated form, whereas the remaining clones were not significantly different than their ancestor (Fig 3D and S5C Fig). These results reinforce that different adaptive paths had been taken by the individual evolved lines.

### Independent *Ylip-HSP90* clones evolved through different adaptive trajectories

**Fig 4:**
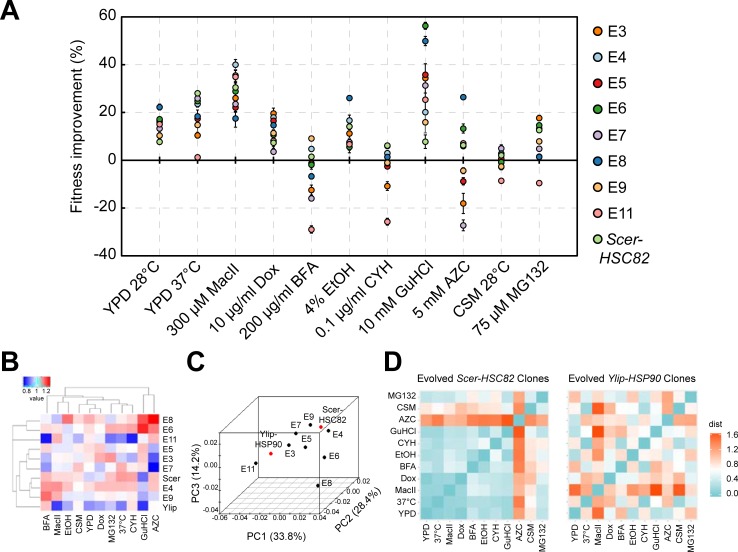
Evolved *Ylip-HSP90* clones exhibit diverged adaptive phenotypes in different conditions. (A) Fitness improvements of the evolved *Ylip-HSP90* clones under 11 different growth conditions, many of which challenge different aspects of protein homeostasis. Cells were grown in liquid cultures and growth rates were measured by plate readers. Error bars are standard errors, *N* ≥ 3. Unless specified otherwise, cells were grown at 28°C. Fitness improvement was calculated by comparing the fitness of the evolved (or *Scer-HSC82*) clone with that of the ancestral *Ylip-HSP90* strain. (B) Hierarchical clustering of the fitness values (i.e., growth rates) reveals divergent evolution of the *Ylip-HSP90* clones. For many of the conditions, some evolved *Ylip-HSP90* clones display enhanced fitness compared with *Scer-HSC82* clones. Individual fitness values were divided by the mean of all fitness values in the same condition to normalize the fitness data to a similar scale between conditions. The color code represents the normalized fitness value (red: high fitness; blue: low fitness). (C) PCA of the fitness values shows that all *Ylip-HSP90* clones evolved diverged phenotypes, scattered across the three principal component dimensions. Explanatory power is shown in brackets next to each principal component. (D) The evolved phenotypes of *Ylip-HSP90* clones are more diverse than those of *Scer-HSC82* clones. Pearson correlation distance (d = 1 − Pearson correlation coefficient) was used to measure whether the evolved clones displayed similar fitness trends under different conditions. A smaller distance indicates a higher similarity between the two conditions. In general, phenotypic distances for evolved *Ylip-HSP90* clones were significantly greater than those of evolved *Scer-HSC82* clones (*p* = 0.001, one-tailed Wilcoxon rank-sum test), suggesting that evolved *Ylip-HSP90* clones have more divergent phenotypes. The color code represents the Pearson correlation distance value (cyan: high similarity; red: low similarity). The numerical data used in the figure are included in S1 Data. See also S6 Fig. AZC, azetidine-2-carboxylate; BFA, brefeldin A; CSM, complete supplement mixture; CYH, cycloheximide; Dox, doxycycline; EtOH, ethanol; GuHCl, guanidine hydrochloride; MacII, macbecin II; PC, principal component; *PCA*, principal component analysis; *Scer*, *Saccharomyces cerevisiae*; *Ylip*, *Yarrowia lipolytica*; YPD, Yeast extract-Peptone-Dextrose.

Both the Hsp104 aggregation assay and our cell morphology measurements suggested that the phenotypes of evolved *Ylip-HSP90* lines are diverged from each other and from their ancestor and *Scer-HSC82* cells. To obtain a more complete picture of the evolved phenotypes, we subjected the evolved clones to two growth media and nine different stress conditions that challenge different aspects of protein homeostasis and then measured their fitness (see Materials and methods). Our results revealed a broad spectrum of adaptive phenotypes in the evolved clones (Fig 4A and S2 Table) so that, even if some strains shared similar fitness levels for one or two conditions, their fitness levels differed under other conditions. Moreover, for many of the conditions we tested, the evolved clones exhibited higher fitness than *Scer-HSC82* cells (S2 Table and Fig 4A). We also tested whether the observed fitness was correlated with the protein abundance of Hsp90 in evolved *Ylip-HSP90* clones. No significant correlation was observed in any tested condition (S3 Table).

To obtain a more general overview of the evolved features in different clones, we performed hierarchical clustering and principal component analysis (PCA) according to their fitness measurements under the 11 tested conditions (Fig 4B and 4C, S6A Fig and S4 Table). Hierarchical clustering revealed that clones E4 and E9 were more similar to *Scer-HSC82* cells than other evolved clones. The first principal component (explaining 33.8% of the variance) of our PCA showed that most clones evolved toward the *Scer-HSC82* phenotype (S6A Fig). Nonetheless, they were not closely clustered. Phenotypic divergence between the evolved clones was further revealed by the second and third principal components (explaining 28.4% and 14.2% of the variance, respectively). These results suggest that *Ylip-HSP90* lines have evolved diverged phenotypes that both differ from each other and from ancestor and *Scer-HSC82* cells.

The observed phenotypic variation in the evolved *Ylip-HSP90* clones suggests that after the heterologous Hsp90 was introduced, different Hsp90-related functions in individual evolving lines were modified to regain fitness. Under this hypothesis, high phenotypic variation should not occur in the evolved *Scer-HSC82* clones (i.e., the control lines), because the network hub has not changed. We performed two types of analysis to address this issue.

We first measured the fitness of evolved *Scer-HSC82* clones under the same 11 growth conditions (S6B Fig) and calculated the variance of fitness improvements among evolved *Ylip-HSP90* and *Scer-HSC82* clones in individual conditions. We then compared the variance between evolved *Ylip-HSP90* and *Scer-HSC82* groups. The data show that the evolved *Ylip-HSP90* clones exhibited greater variance than the evolved *Scer-HSC82* group (*p*-value = 0.039, one-tailed Student *t* test), indicating that responses of evolved *Ylip-HSP90* clones to different stress conditions were more varied than evolved *Scer-HSC82* clones (S6C Fig).

In the second analysis, we calculated the Pearson correlation distances between fitness levels under different conditions (see Materials and methods, S5 Table) to quantify the variation in distribution patterns of individual fitness between each pair of conditions. If individual clones show similar ranking patterns for fitness between two conditions, Pearson correlation distances will be small. In contrast, large Pearson correlation distances indicate that individual evolved clones behave very differently in two conditions. Overall, pairwise comparisons revealed that the evolved *Scer-HSC82* clones behaved similarly under different stress conditions, except for the complete supplement mixture (CSM) and azetidine-2-carboxylate (AZC) media (Fig 4D and S5 Table). The stress condition–based phenotypic distance of evolved *Scer-HSC82* clones is significantly smaller than that of evolved *Ylip-HSP90* clones (S6D Fig), supporting our hypothesis that high phenotypic variation only occurred in evolved *Ylip-HSP90* clones.

### Most mutations in evolved *Ylip-HSP90* clones are associated with various Hsp90-related functions

To explore the genetic basis of the evolved phenotypes, we sequenced the genomes of the evolved *Ylip-HSP90* clones. On average, each evolved clone hosted eight protein-sequence-changing mutations (seven nonsynonymous and one insertion/deletion) and 14 total mutations (S7 Fig). The calculated mutation rate (5.45 × 10^−10^ per bp per generation) is comparable with the estimated spontaneous mutation rates from previous studies [38, 39]. Only a small group of genes were recurrently mutated in multiple evolved clones (i.e., *BRE5* in three clones and *BUD2*, *FMP30*, *SIR3*, and *HXK2* in two clones, S6 Table). We investigated the *BRE5* gene further in the reconstitution experiments. In evolved clones, we found that large-scale copy number variation only occurred in mitochondrial genomes and ribosomal DNA, and that small-scale copy number variation was also rare outside subtelomeric regions (S7 Table).

**Fig 5:**
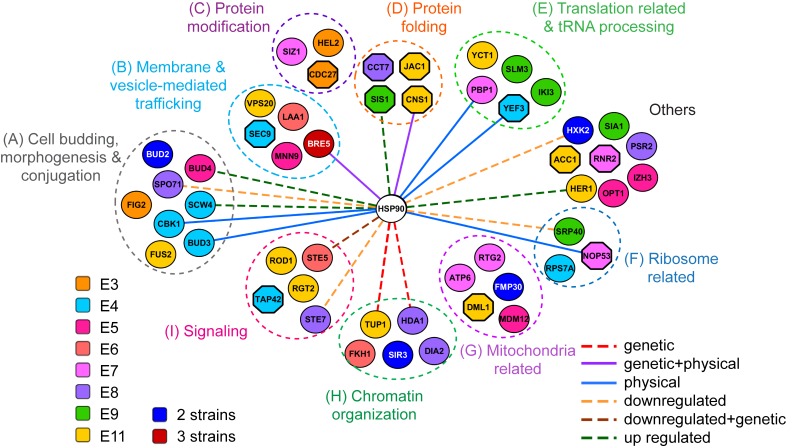
Evolved *Ylip-HSP90* clones contain mutations in the genes involved in Hsp90-related functions. Most mutant genes identified in evolved *Ylip-HSP90* clones can be grouped into several Hsp90-related functions, and about one third of them (32%) are Hsp90 interactors or Hsp90-dependent proteins (connected to Hsp90 by lines). Essential genes in *Saccharomyces cerevisiae* are displayed as octagonal nodes. The evolved clones containing the recurrent mutant genes are listed as follows: *BRE5* in E3, E5, and E11; *BUD2* and *FMP30* in E3 and E9; *HXK2* in E3 and E6; and *SIR3* in E8 and E11. *Ylip*, *Yarrowia lipolytica*.

When the gene functions of all identified protein-sequence-changing mutations were analyzed further (Table 1 and S8 Table), we found that most of them could be categorized into several Hsp90-related functions [14, 40], including protein synthesis, folding and modification, intracellular trafficking, ribosome biogenesis, cellular conjugation, chromatin organization, and signaling, as well as mitochondrial physiology (Fig 5). Many of these genes have known interactions with Hsp90 or belong to the Hsp90-dependent proteome [17, 41], raising the possibility that evolved yeasts might adapt by modifying different Hsp90 subnetworks.

**Table 1:** Enriched gene ontology (GO) categories of the protein-sequence-changing mutations in evolved *Ylip-HSP90* clones.

GO categories	Genes	*p*-value	Enrichment score	Functional group
Conjugation	BUD3, FIG2, STE7, STE5, SCW4, FUS2, ROD1	1.29 × 10^−4^	7.21	A
Invasive growth in response to glucose limitation	TUP1, FIG2, STE7, STE5, DIA2	1.29 × 10^−4^	10.89	
Cell budding	BUD3, BUD4, BUD2, CBK1	1.23 × 10^−3^	8.67	A
Response to chemicals	TUP1, FIG2, RGT2, STE7, STE5, RTG2, FKH1, FUS2, SIS1, ROD1	6.37 × 10^−3^	2.84	
Cell morphogenesis	FIG2, FUS2	1.28 × 10^−2^	7.80	A
Protein modification by small protein conjugation or removal	CDC27, HEL2, SIZ1, BRE5, DIA2	1.50 × 10^−2^	3.77	C
Chromatin organization	TUP1, HEL2, RTG2, FKH1, SIR3, HDA1, DIA2	1.54 × 10^−2^	2.80	H
Signaling	RGT2, STE7, STE5, RTG2, TAP42, ROD1	1.54 × 10^−2^	3.11	I
Nuclear transport	RTG2, SRP40, SIS1, ACC1, NOP53	1.54 × 10^−2^	3.50	
Cytokinesis	BUD3, BUD4, BUD2	1.73 × 10^−2^	4.54	A
Regulation of transport	TUP1, BRE5, ROD1	1.73 × 10^−2^	4.54	
Protein folding	CNS1, CCT7, SIS1	2.35 × 10^−2^	3.93	D
Regulation of protein modification process	STE7, STE5, SIZ1	3.72 × 10^−2^	3.47	
Nuclear organization	FUS2, ACC1	4.64 × 10^−2^	3.90	
Ion transport	ATP6, YCT1, VPS20, MDM12, SIA1	4.86 × 10^−2^	2.39	

List of significantly enriched GO categories (*p*-value < 0.05 after the Benjamini-Hochberg correction) among the genes with protein-sequence-changing mutations. Enrichment scores were calculated by dividing the proportion of mutated genes classified into the indicated category by the proportion of those in the genome background. The GO categories overlapped with the Hsp90-related functional groups (Fig 5) are labeled.

Abbreviations: GO, gene ontology; *Ylip*, *Yarrowia lipolytica*.

### Segregant analysis identifies beneficial mutations with strong fitness effects

Not all the mutations in the evolved clones are likely to contribute equally to the adaptive phenotypes. We performed segregant analysis to map the mutations with strong effects in three evolved clones (E4, E5 and E7). These clones were selected because E4 and E5 clones showed opposite trends in cell morphology, and E7 has the largest improvement in protein homeostasis (Fig 3). The evolved *Ylip-HSP90* clones were backcrossed to the ancestral *Ylip-HSP90* strain and their F1 spores (from 40–45 four-viable spore tetrads) were analyzed. In all three evolved clones, our genetic analysis indicated that multiple loci were involved in the evolved phenotypes (S9 Table). The F1 progeny with phenotypes similar to evolved or ancestral clones were grouped into good and bad spore pools, respectively (see Materials and methods). Genomic DNA was isolated from both pools and then subjected to Illumina sequencing.

Based on our computational simulation, two criteria were used to select the mutations with strong effects: (1) the mutation frequency in the good spore pool is greater than 70% or (2) the difference in mutation frequencies between good and bad spore pools is greater than 45%. Nine mutations—*sec9-E310K* and *tap42-F72S* from E4; izh3-S115* (* indicating a premature stop codon), *bud4-P981fs* (*fs* indicating a frameshift mutation), *bre5-L433F*, and *mdm12*-*F148S* from E5; and *siz1-P401L*, *pbp1-N318fs*, and *nop53-E289_N290insE* (*ins* indicating an insertion) from E7 (S10 Table)—were identified and used for reconstitution experiments to confirm their effects.

### Individual mutations exert condition-specific effects

**Fig 6:**
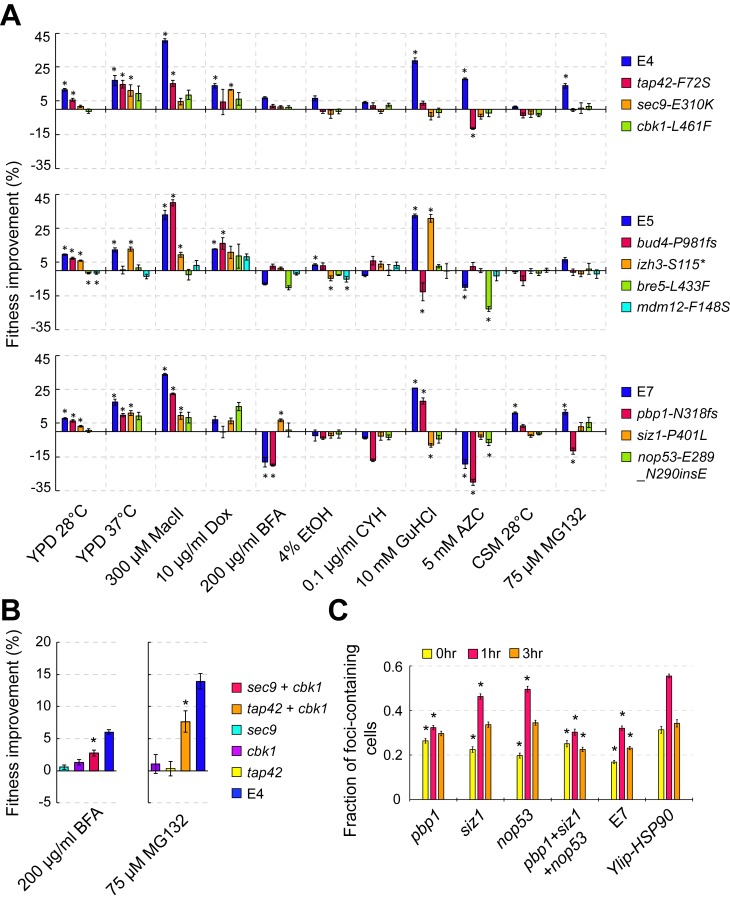
Allele replacement confirms the contribution of the mapped mutations in evolved phenotypes. (A) Fitness improvements of the reconstituted clones under 11 growth conditions. Nine mapped strong-effect mutations and *cbk1-L461F* were reintroduced into the ancestral *Ylip-HSP90* strain using the CRISPR/Cas9 system, and the fitness of reconstituted clones was then compared with that of the ancestral *Ylip-HSP90* strain. Cells were grown in liquid cultures and growth rates were measured by plate readers. Individual mutations exerted dominant effects in at least one condition, and antagonistic pleiotropy under different stress conditions was widely observed. Error bars are standard errors, *N* ≥ 3. Asterisks indicate that the fitness values are significantly different from the ancestral *Ylip-HSP90* strain (*p* < 0.05 after the Benjamini-Hochberg correction, two-tailed Wilcoxon rank-sum test). See also S8 Fig. (B) Positive genetic interactions between *cbk1-L461F* and the other two mutations result in the fitness improvement not observed in single mutants when grown in media containing BFA or MG132. Error bars are standard errors, *N* ≥ 4. Asterisks indicate that the fitness of double mutants is significantly greater than that of single mutants (*p* < 0.05 after the Benjamini-Hochberg correction, two-tailed Wilcoxon rank-sum test). (C) All three single mutations in the E7 clone reduce the formation of Hsp104 foci at 37°C, but *pbp1-N318fs* has the strongest effect. Each measurement was compared with that of ancestral *Ylip-HSP90* cells from the same time point, and asterisks indicate a *p*-value <0.01 after the Benjamini-Hochberg correction, according to one-tailed Wilcoxon rank-sum test. *N* ≥ 14. Error bars are standard errors. The numerical data used in the figure are included in S1 Data. AZC, azetidine-2-carboxylate; BFA, brefeldin A; CRISPR/Cas9, clustered regularly interspaced short palindromic repeats/CRISPR-associated protein 9; CSM, complete supplement mixture; CYH, cycloheximide; Dox, doxycycline; EtOH, ethanol; GuHCl, guanidine hydrochloride; *L461F*, leucine 461 to phenylalanine; MacII, macbecin II; *N318fs*, asparagine 318 frameshift; *Ylip*, *Yarrowia lipolytica*; YPD, Yeast extract-Peptone-Dextrose.

Our phenotypic assays had revealed that independent *Ylip-HSP90* lines evolved diverse phenotypes (Fig 4). To understand how the evolved mutations contribute to the broad spectrum of phenotypes, we used the clustered regularly interspaced short palindromic repeats/CRISPR-associated protein 9 (CRISPR/Cas9) system to reintroduce these mutations into the ancestral *Ylip-HSP90* strain and examined the fitness of these mutants under different stress conditions (Fig 6A, S8 Fig and S11 Table). Four major conclusions can be drawn from the phenotypic assays. First, individual mutations usually had dominant effects under at least one stress condition (e.g., in clone E7, the *siz1*, *pbp1*, and *nop53* mutants exhibited strongest effects under conditions of 37°C, macbecin II treatment, and doxycycline treatment, respectively), confirming that these mutations really contribute to the evolved phenotypes. Second, several mutations exhibited positive effects under one stress condition but negative effects under others (e.g., in clone E7, *pbp1* mutants had a positive effect under macbecin II treatment but a negative effect under the MG132 stress condition), indicating that antagonistic pleiotropy is common among the evolved mutations. Third, in a few stress conditions (e.g., for clone E5 in medium containing macbecin II), the sum of individual effects exceeded the effect observed in the evolved clone, suggesting the existence of negative epistasis between mutations. Fourth, for some other conditions (e.g., clone E4 in medium containing MG132), the sum of all individual effects was still far below the effect observed in evolved clones. This outcome can be explained if there is positive epistasis between mutations. Alternatively, some mutations with weak individual effects but that are strongly epistatic to other mutations might have been missed by our mapping exercise. We selected one mutation from the E4 clone, *cbk1-L461F*, that exhibited a mutation frequency slightly lower than our segregant analysis criteria to test these possibilities. The *cbk1-L461F* single mutation did not result in any significant effects under any tested stress condition (Fig 6A). However, when *cbk1-L461F* was combined with *sec9-E310K* or *tap42-F72S* and tested in all conditions, it displayed positive epistatic effects with *sec9-E310K* and *tap42-F72S* in medium containing brefeldin A (BFA) and MG132, respectively (Fig 6B and S11 Table).

In addition to its fitness improvement, our Hsp104 aggregation assay showed that the E7 clone has a strong aggregate clearance phenotype (Fig 3C). This allowed us to characterize the contribution of individual mutations to protein homeostasis. We found that all three single mutations (*siz1*, *pbp1*, and *nop53*) reduced the formation of Hsp104 foci at 37°C, but *pbp1-N318fs* had the strongest effect (Fig 6C). When all three mutations were combined in a single clone, the further enhanced aggregate clearance phenotype was observed at the 3-hour time point. These results reveal a specific role of *pbp1-N318fs* in maintaining protein homeostasis under heat stress conditions.

## Discussion

The Hsp90 network performs many crucial functions in eukaryotic cells and probably represents a robust system resilient to genetic perturbations [16, 40]. Nevertheless, the ability of Ylip-Hsp90 to enhance hypersaline tolerance in *S*. *cerevisiae* cells and the compromised fitness of such cells under other stress conditions indicate that even a central hub like Hsp90 can change its functions or partners during evolution. This seeming discrepancy may be explained by a computational simulation study of complex signaling circuits that showed phenotypic robustness and variability are positively correlated [42]. Because a broad range of genotypes usually supports a robust phenotype, a greater diversity of phenotypes is accessible from among the genotypic neighborhood. This model implies that a robust system like the Hsp90 network can still be genetically adjusted to facilitate adaptation to novel environments. If the Hsp90 network is continually modified in a specific direction (e.g., to tolerate hypersaline stress), it may eventually lead to alterations in a network hub such as Ylip-Hsp90.

Evolvability represents an organism’s capacity to generate selectable phenotypic traits during evolution [43], so the level of evolvability may influence the likelihood of an organism surviving in or adapting to a new habitat. In developmental biology, large phenotypic changes or innovations are often associated with changes in the transcriptional network hubs [44–46]. Despite strong evidence supporting a link between network hubs and evolvability, it is difficult to study directly the effect of changed network hubs on evolutionary trajectories using natural populations. Our laboratory evolution experiments provide an alternative approach to address this issue. In the current study, we replaced the Hsp90-coding gene of *S*. *cerevisiae* with the ortholog from a distant species. Nonetheless, a similar evolutionary process may occur in nature under the following conditions: (1) a hybrid progeny carries incompatible gene pairs that compromise the function of a network hub. Genetic incompatibility has been observed to exist between closely related species and cause defects in different important cellular functions [47]. Although these types of hybrids have lower fitness and are likely to be eliminated in most cases, they may also create the chance for the organism to evolve new phenotypes, especially in the hybrid zone where hybrids are constantly generated. (2) Mutations occur in critical domains of a hub gene. Mutagenesis experiments in Hsp90 have shown that even a single amino acid change can alter the activity or interacting partners of the protein completely [48]. Such mutations will often result in the low fitness of the cell, as in the hybrid case. However, they may lead to the evolution of new phenotypes if compensatory mutations that restore fitness occur and are subsequently fixed by selection [49]. (3) A multifunctional gene is obtained through horizontal gene transfer (HGT). In recent years, HGT has been shown to occur frequently, even in eukaryotes [50, 51]. A foreign multifunctional protein may disturb the original cellular network and open a new evolutionary path.

Network structures contribute to phenotypic robustness against genetic or environmental perturbations [52]. When network hubs are compromised, pre-existing cryptic genetic or nongenetic variation may be exposed that facilitates short-term population adaptation [7]. Our study reveals a different role played by network hubs in long-term evolution in a population without much genetic variation. *S*. *cerevisiae* cells immediately gained hypersaline tolerance like *Y*. *lipolytica* cells solely by replacing a single hub gene, *HSP90*, with the corresponding ortholog. This is similar to a previous observation that bacteria dramatically increased the growth rate at lower temperatures with the introduction of psychrophilic chaperonins [53]. Moreover, the novel Hsp90 allowed individual clonal populations to evolve diverse phenotypes unobserved in cells hosting native Scer-Hsc82. These novel phenotypes may enable evolved cells to explore niches inaccessible to Scer-Hsc82–hosting cells and, in the long term, may broaden opportunities for population diversification and speciation.

Previous laboratory evolution experiments in bacteria showed that when essential ribosomal genes or translation factors were replaced by their orthologs from other bacteria or the ancestral sequence, expression levels of the introduced orthologous genes were often amplified to compensate for the incompatibility [54–56]. In our evolved clones, protein levels of Ylip-Hsp90 were not changed, suggesting that the cells were able to quickly gain other mutations to ameliorate the growth defect in the conditions under which they evolved. One major function of network hubs is to coordinate different subnetworks (or pathways) so that the cell can quickly respond to environmental changes. However, when a hub is compromised or altered (such as in the Ylip-Hsp90–hosting cells), any mutation that can improve cell fitness under the existing growth conditions will be selected, even if it has costs in other conditions or distorts the balance between different subnetworks. The evolved *Ylip-HSP90* clones restored only some of the functions of Hsp90 despite that all clones had similar or higher fitness in rich medium compared to *Scer-HSC82* cells (Figs 3C, 3D and 4).

By sequencing the evolved clones and reconstituting mutations, we confirmed that individual subnetworks of Hsp90 had been modified in different evolved lineages. As expected, some of the evolved mutations showed strong antagonistic pleiotropy, exhibiting improved cell fitness in rich medium but growth defects under other stress conditions. Because cells are constantly challenged by environmental fluctuations, it is likely that another layer of compensatory mutations can be selected to resolve the fitness conflict of pleiotropic mutations under different conditions [57]. These compensatory mutations may not exhibit adaptive effects by themselves but will be epistatic to the previous pleiotropic mutations. This hypothesis is indirectly supported by our reconstitution experiments, which showed that intergenic epistasis is common among the evolved mutations. After obtaining compensatory mutations, modified subnetworks are likely to stay in the lineage and to have a long-term influence on evolutionary paths.

**Fig 7:**
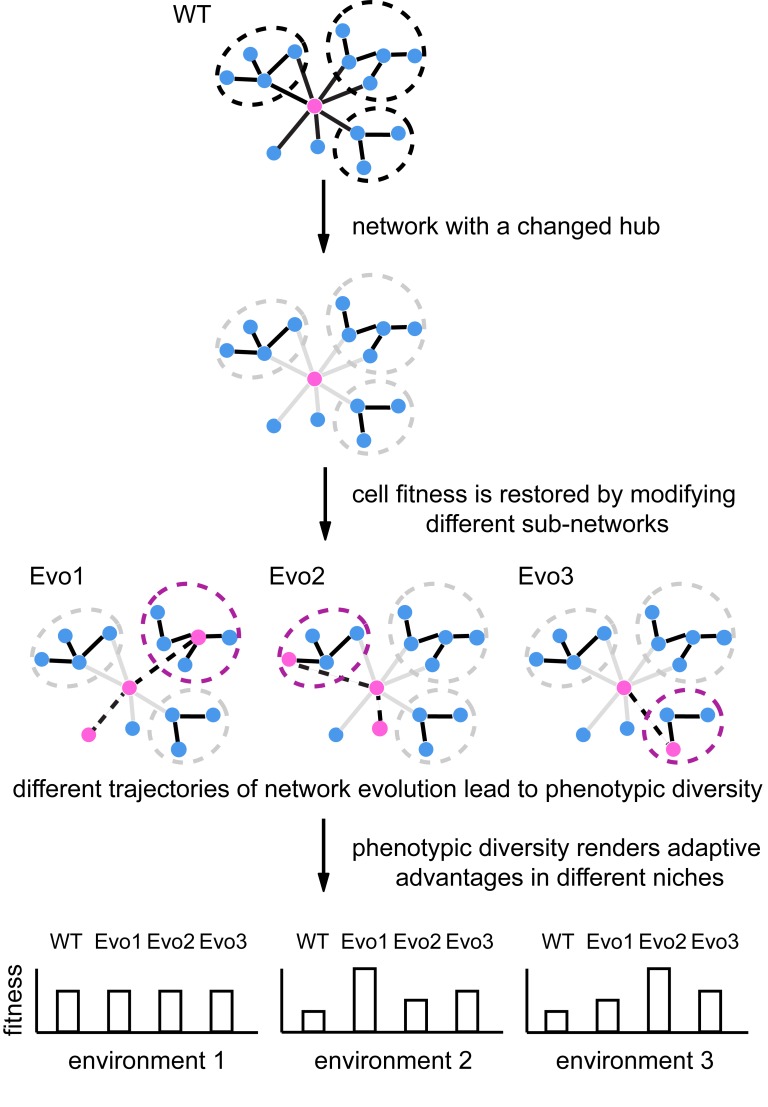
A model showing how a changed network hub allows the cell to reshape the network structures in different directions to broaden its evolutionary potential. When a network hub is altered, cells regain fitness by modifying different parts of subnetwork structures. Such events allow cells to pursue different evolutionary paths that are inaccessible to wild-type cells. Evo, evolved line; WT, wild type.

The fact that no mutations occurred in the *Ylip-HSP90* gene indicates a strong constraint on the hub, probably due to pleiotropic effects. Therefore, evolved cells resolved the fitness conflict through modifying the functions or structures of some subnetworks. Similar processes have been observed in the evolution of transcription networks. Although different species may share the same transcription factors, the downstream targets of the orthologs could have been rewired greatly [58, 59]. Previous studies of digital organisms have suggested that deleterious mutations might act as stepping stones for the evolution of complex phenotypes inaccessible to the wild type [60, 61]. Our study provides direct evidence that diverse phenotypes can evolve when an essential network hub is altered. Robust networks are often connected to a larger phenotypic space [42]. An initial adaptation to a changed or compromised network hub allows a cell to reshape its network structures in different directions, which further broadens its evolutionary potential (Fig 7).

## Materials and methods

### Construction of the HSP90 replacement strains

All the *S*. *cerevisiae* strains were derived from R1158 (JYL1821) containing the tTA transactivator with the CMV promoter that allows us to down-regulate the expression level of the *TetO*_*7*_ promoter–containing gene by adding doxycycline [62]. In *S*. *cerevisiae*, the essential function of Hsp90 is performed by two highly similar and functionally redundant genes, *HSC82* and *HSP82* (protein identity and similarity are 0.969 and 0.987, respectively) [9]. We first generated the HSP90 plasmid shuffling strain (JYL1827) in which both *HSC82* and *HSP82* were deleted, and the essential Hsp90 functions were performed by an *HSC82*-containing plasmid (pRS416-*Scer-HSC82*). The coding regions of *HSC82* and its orthologs were PCR amplified from the genomic DNA or cDNA of *S*. *cerevisiae*, *N*. *castellii*, *K*. *lactis*, or *Y*. *lipolytica* and cloned into a plasmid (pRS413-pTetO_7_) containing the *kan*^*R*^*-TetO*_*7*_*-TATA*_*CYC1*_ cassette from RP188 [63]. These ortholog-containing plasmids were then transformed into the shuffling strain to replace pRS416-*Scer-HSC82*. The ortholog-replacement strains were checked using PCR to confirm the loss of pRS416-*Scer-HSC82* and are denominated as JYL5001 (*Scer-HSC82*), JYL1831 (*Ncas-HSP90*), JYL1833 (*Klac-HSP90*), and JYL1835 (*Ylip-HSP90*). In our experiments, the orthologs of Hsp90 were all carried by the plasmid so they could be easily shuffled to check whether the observed effects are ortholog specific.

### Experimental evolution

For the evolution experiment, JYL5001 and JYL1835 were transformed with HindIII-linearized GFP (pGS62) and DsRED (pGS64) integration plasmid DNA [64] to generate green and red fluorescent protein-labeled ancestral strains. We mixed the two colored subpopulations of *Ylip-HSP90* or *Scer-HSC82* cells in an approximate ratio of 1:1 to establish starting cultures and then divided them into 12 evolving and 12 control lines. These cell lines were grown in YPD medium at 28°C through a daily 1,000-fold dilution (about 10 generations) with an effective population size of 4.69 × 10^5^ cells on the first 140 days and 2.79 × 10^6^ cells after day 140. The effective population size was estimated using the formula, *N*_*e*_ = *N*_*0*_ × *g*, in which *N*_*0*_ is the initial population size and *g* is the number of generations during a single cycle of growth [65]. We tracked the proportion of the green and red subpopulations in each lineage using the BD LSR II system (Becton Dickinson, Franklin Lakes, NJ). Fixation of the evolved culture is defined when either the green or red subpopulation reaches a population frequency of 95%.

### Cell fitness assays

For the fitness spot assay on plates, around 10^3^ cells were taken from overnight cell cultures growing in YPD at 28°C and spotted onto the plates following 10-fold serial dilutions. Plates were then incubated at specified temperatures until individual colonies became visible. Colony size and cell density were used to estimate the cell fitness and survival rate for each condition. For the fitness assay in liquid, overnight cell cultures growing in YPD at 28°C were inoculated into 120 μL fresh medium in 96-well tissue culture plates at an initial concentration of 0.1 OD_595_, and cell densities (OD_595_) were measured every 10 minutes using Infinite 200 series plate readers (Tecan, Mannedorf, Switzerland) with a shaking cycle (1 minute shaking and 3 minutes standing) until the culture reached diauxic shift. Growth rates (OD_595_/hour) of the sample were calculated using the 10-point sliding window, and the maximal growth rate was used to represent the fitness value. Fitness improvement compared with ancestors was calculated as (F_x_/F_a_−1) × 100%, where F_x_ is the fitness value of evolved or reconstituted clones and F_a_ is the fitness value of the ancestral strain.

In order to uncover the functional differentiation between Hsp90 orthologs, the ortholog-replacement strains were examined under YPD at 28°C and 16 other conditions, including 0.015% or 0.03% H_2_O_2_, 150 mM MgCl_2_, 50 μM CH_3_COOH, 0.3 μM tunicamycin (cat. no. T7765, Sigma-Aldrich, St. Louis, MO), 5 mM guanidine hydrochloride (GuHCl), pH 5 or pH 9, 1 μg/mL doxycycline (cat. no. D9891, Sigma-Aldrich), 0.5 or 1 M NaCl, 0.2 M LiCl, 16°C, 34°C, or 37°C, and 1.8 M sorbitol. Chemicals were added into YPD to prepare media for specific conditions. Liquid fitness assays were used in all measurements.

To characterize the evolved phenotypes, we measured the fitness of evolved clones under nine different stress conditions: AZC (cat. no. A0760, Sigma-Aldrich), a proline analog that can be incorporated into newly synthesized proteins and cause failure in folding or structural instability [66, 67]; cycloheximide (CYH, cat. no. C7698, Sigma-Aldrich), which inhibits the 80S ribosome from translating mRNA into proteins [68]; BFA (cat. no. B6542, Sigma-Aldrich), which blocks protein trafficking and affects the central vacuolar secretory pathway, resulting in ER/Golgi membrane mixing [69] and an unfolded protein response [70]; high temperature (37°C) and ethanol (EtOH), which induce protein misfolding and change the membrane protein composition [71]; doxycycline, which reduces the expression level of Hsp90 orthologs; GuHCl, which inhibits the disaggregase activity of Hsp104 and reduces cell thermotolerance [72]; MG132 (cat. no. C2211, Sigma-Aldrich), a compound that inhibits protein degradation by blocking proteasome activity [73]; and macbecin II (MacII, cat. no. NSC 330500, NIH, Bethesda, MD), which is a compound that inhibits the ATPase activity of Hsp90. CSM at 28°C was a suboptimal condition. Apart from CSM, as well as MG132 that was added to synthetic medium (0.17% yeast nitrogen base without ammonium sulfate supplemented with 0.1% proline, amino acids, 2% glucose, and 0.003% SDS) [74], all these chemicals were added into YPD medium. Liquid fitness assays were used in all measurements.

### Western blotting for measuring the abundance of Hsp90

We constructed pRS413-*pTetO*_*7*_*-TAP-Scer-HSC82* and pRS413-*pTetO*_*7*_*-TAP-Ylip-HSP90* plasmids, in which a tandem affinity purification (TAP) tag was fused to the N-terminus of the Hsp90 protein. These plasmids were then transformed into corresponding ancestral or evolved clones. Cell pellets were collected by centrifugation, washed once with ddH_2_O, and lysed using the NaOH-lysis method [75]. The anti-TAP-tag antibody (cat. no. CAB1001, Thermo Fisher Scientific, Waltham, MA) was used to detect TAP-Scer-Hsc82 or TAP-Ylip-Hsp90 proteins, and the anti-G6PDH antibody (cat. no. A9521, Sigma-Aldrich) was used to detect the G6PDH protein as the internal control.

### V-src kinase assay

Ancestral *Scer-HSC82* and *Ylip-HSP90* strains were first transformed with the plasmid Y316v-srcv5 containing v-src with a C-terminal V5 tag under a galactose-regulated promoter [76]. Cells were then maintained on 2% glucose CSM-Ura plates to prevent the expression of v-src. Before the experiment, cells were grown to log phase in 2% glucose CSM-Ura liquid cultures, transferred to 2% raffinose CSM-Ura, and then grown to about 5 × 10^6^ cells/mL. Cells were pelleted, resuspended in 2% galactose CSM-Ura, and grown for 6 hours to induce the expression of v-src. The whole experiment was carried out at 28°C. Cell pellets were collected by centrifugation, washed once with water, and lysed with the NaOH-lysis method. Total protein extracts were mixed with the sample buffer (50 mM Tris, pH 6.8, 2% SDS, 10% glycerol, 0.004% bromophenol blue, and 2% 2-Mercaptoethanol) and boiled at 100°C for 5 minutes. The anti-phospho-tyrosine antibody 4G10 (cat. no. 05–321, Merck Millipore, Burlington, MA) was used to detect phosphorylated tyrosine residues, and the anti-V5-Tag antibody (cat. no. MCA1360, BioRad, Hercules, CA) was used to detect the v-src protein.

### Flow cytometry of DNA content

Flow cytometry was used to determine the DNA content of ancestral and evolved cells. Total 5 × 10^6^ log-phase cells were harvested, resuspended in ice-cold fixation buffer (40% ethanol, 0.1 M sorbitol, and 5 mM EDTA), and then kept at −20°C overnight. The fixed cells were then washed twice with 1 mL ddH_2_O and then washed with 1 mL PBS + 0.5% Triton X-100. After washing, the cells were treated with 0.5 mL 50 mM Tris-Cl (pH 8.0) with 150 μg/mL RNase A and incubated at 37°C for 16–18 hours. SYTOX Green nucleic acid stain (Invitrogen, Carlsbad, CA) and 38 mM sodium citrate were mixed in a 1:800 ratio, and 300 μL of the mixture was added into the cell solution. The stained cells were diluted into 1 mL 0.1 M EDTA and sonicated for at least 3 minutes prior to flow cytometry. A total of 10,000 cells were scored for DNA content using the BD FACScan system (Becton Dickinson). The E10 clone was excluded from further examination because of its altered ploidy.

### Hsp104 aggregation assay

A blue fluorescence protein (BFP) cassette was amplified from the pFA6a-link-yomTagBFP2-Kan plasmid and integrated into the genome to generate an Hsp104-BFP fusion protein in all tested strains [77]. Log-phase cells growing in YPD at 25°C were transferred to a 37°C water bath and cells were collected at 0, 1, 2, and 3 hours after the temperature shift. The samples were resuspended in 200 μL PBS and transferred to a Glass Bottom ViewPlate-96F (PerkinElmer, Waltham, MA) that was precoated with concanavalin A (cat. no. C2010, Sigma-Aldrich). Images of Hsp104-BFP foci were acquired using an ImageXpress Micro XL system (Molecular Devices, San Jose, CA) and analyzed using a custom-built module under MetaXpress High-Content Image Acquisition and Analysis Software (Molecular Devices).

### Cell morphology assay

Log-phase cells were harvested and washed twice with PBS, and cell walls were stained with either 25 μg/mL NHS-Rhodamine (E3, E5, E7, E8, E9, green *Ylip-HSP90*, and *Scer-HSC82* ancestors) or NHS-Fluorescein (E4, E6, E11, and red *Ylip-HSP90* and *Scer-HSC*82 ancestors) (Pierce, Rockford, IL) for 8 minutes in the PBS buffer with 0.1 M NaHCO_3_ and 5 mM EDTA. Cells were then washed twice with PBS and sonicated for 3 minutes before 2 × 10^4^ cells were transferred to a Glass Bottom ViewPlate-96F for image acquisition. We used Calmorph to calculate the ratio of the long versus short axes of yeast cells (http://scmd.gi.k.u-tokyo.ac.jp/datamine/calmorph/) [78].

### Hierarchical clustering and PCA

For hierarchical clustering, we divided individual fitness values by the mean of all fitness values for the same stress condition to normalize fitness data to a similar scale between conditions. We applied a hierarchical clustering method in R (v3.4.0) [79] to group the evolved Ylip-Hsp90-hosting clones according to their fitness similarity between different conditions. The strain and condition dendrograms were constructed with the hclust function and the heatmap was drawn with the heatmap.2 function in the gplots R package [80]. For PCA, fitness values were used directly, i.e., without normalization, and the prcomp function in R was used to conduct the analysis.

### Calculation of Pearson correlation distances

We used the growth rates of all evolved *Scer-HSC82* and *Ylip-HSP90* clones to conduct pairwise Pearson correlations between each condition. The Pearson correlation distance (d = 1 − Pearson’s correlation coefficient) is used to represent the phenotypic distance between each pair of conditions. A large d means the fitness distributions of evolved clones have very different patterns in two conditions. We used the fviz_dist function of the factoextra package in R to draw the heatmap [81].

### Whole genome sequencing analysis

Yeast genomic DNA was isolated using the previously described phenol/chloroform method with one more run of 200 μL of PCIA resuspension and 95% ethanol precipitation to remove the residual RNase A [82]. Isolated genomic DNA was fragmented using a Covaris microTUBE M220 Focused-ultrasonicator (Covaris, Worburn, MA) and DNA libraries were prepared using a TruSeq DNA PCR-Free HT Library Prep Kit (Illumina, San Diego, CA). Ancestral and each evolved *Ylip-HSP90* clone was sequenced to an average depth of 100-fold coverage with a rapid-mode Illumina HiSeq 2500 system (250 bp paired-end reads) and the spore pools were sequenced to an average depth of 200-fold coverage with the NextSeq 500 system (150 bp paired-end reads).

For single nucleotide polymorphism (SNP) analysis, raw reads were mapped, and SNPs with coverage greater than 20-fold and a frequency higher than 35 (for the segregant analysis, the allele frequency threshold was lowered to 10 to include more low-frequency evolved mutations) were called using CLC bio software (CLC bio, Aarhus, Denmark). Evolved mutations were manually identified by comparing the mutation lists between evolved and ancestral *Ylip-HSP90* clones, and poor-quality SNPs were excluded after manual checking using the Integrative Genome Viewer [83]. Two kinds of mutations were further excluded from our evolved mutation list (S4 Table): double-called SNPs due to overlap between two open reading frames, and SNPs (*grx3-A196V*, *hop1-K510fs*, *isu1-I100V*, and *rsf2-C966G*) shared by multiple evolved clones that were likely to be pre-existing SNPs in the ancestral population.

For copy number variation (CNV) analysis, raw reads were mapped via BWA-MEM and CNVs were analyzed via the SAMtools program [84]. The copy number of each gene was calculated by normalizing gene coverage by the median of whole genome coverage derived from the SAMtools bedcov module. Genes with copy number variation greater than 0.8 compared with the ancestral strain were selected and the fold-change is provided in S7 Table.

### F1 segregant analysis

E4, E5, and E7 clones were backcrossed to the ancestral strain and the diploids were sporulated. Tetrads were dissected and only spores from four-viable spore tetrads were used. The fitness of F1 haploid progeny was examined under the following conditions: YPD at 28°C, YPD at 37°C, and YPD + GuHCl at 28°C. The latter two conditions were included because they had a better resolution to separate the ancestral and evolved phenotypes. F1 haploid progenies with fitness similar to the evolved or ancestral clones were assigned into good or bad spore pools, respectively. A maximum of one good and one bad spore was selected from a single tetrad. If all four progeny from a single tetrad had fitness that deviated from the evolved and ancestral strains, then none were selected. Individual E7 spores behaved similarly under the high temperature and GuHCl conditions (r = 0.851, Pearson correlation). When cells behaved differently under two conditions, we used the more prominent phenotype of the evolved clone to select the spores (i.e., fitness at 37°C for E4 and fitness in GuHCl-containing medium for E5). We picked 5–20 spores for each pool and employed Illumina sequencing to examine the mutant allele frequency (S9 Table).

The two frequency thresholds used to select the mutations with strong effects were determined based on computational simulations following the previous method [85]. The 90%, 95%, and 99% percentiles of 10,000 times of simulations with the coverage of 200 and pool size *N* = 5, 9, 10, 18, and 21 were listed in S10 Table. We selected the threshold frequency that approximated to *N* = 9 as the general criteria for all three analyzed evolved clones.

### Gene ontology enrichment and network analysis

The genes containing protein sequence mutations from all evolved *Ylip-HSP90* clones were subjected to Gene Ontology (GO) analysis using Yeast GO-Slim Process embedded in Saccharomyces Genome Database (SGD: https://www.yeastgenome.org/). GO categories with only one gene were excluded. Enrichment scores (Table 1 and S8 Table) were calculated by dividing the proportion of mutated genes classified into the indicated category by the proportion of those in the genome background. Significance of enrichment was analyzed using hypergeometric test (phyper module) in R [79], and *p*-values were adjusted using the Benjamini Hochberg method [86]. We excluded genes with uncharacterized function or dubious open reading frames, including YLR345W, YEL034C-A, YMR102C, YLR419W, YOL166C, and Q0182.

To understand the functional relationship between the mutated genes and Hsp90, we constructed the functional network between them. The genetic and physical interactions of Hsp90 were first constructed using STRING’s website (https://string-db.org/), and then each interacting pair was confirmed using the information from the SGD (https://www.yeastgenome.org/). We manually incorporated the up- and down-regulated protein-level data from a proteomics analysis [17] to make the regulatory network more complete. Gene functional groups shown in Fig 5 were derived from enriched GO categories and gene-specific description in SGD. The interaction network was constructed with both *S*. *cerevisiae* Hsp90 coding genes and all genes that contain protein-sequence-changing mutations in evolved *Ylip-HSP90* clones using the cytoscape software [87].

### CRISPR/Cas9-mediated mutant reconstitution

Evolved mutations were reconstituted in the ancestral shuffling strain carrying the pRS415-*pTetO*_*7*_*-Ylip-HSP90* plasmid (S12 Table). The CRISPR-Cas9 system was modified from previous methods [88, 89]. The guide RNA expression plasmid pRS426-gRNA-AB was modified from pRS426-gRNA.CAN1.Y [88], with BamHI and EcoRI cutting sites added to both sides of the gRNA sequence.

pRS426-gRNA-AB was linearized by BamHI and EcoRI digestion. Guide RNA was amplified by two-fragment fusion PCR using four primers: IgRNAAB-F, target gene gRNA-R, target gene gRNA-F, and IgRNAAB-R (S12 Table). Donor DNA sequences were around 300–600 bp in length and were PCR amplified from the evolved clones. The original plasmid carrying the high-specificity Cas9 nuclease, eSpCas9 (1.1) [90], was obtained from Addgene (plasmid number 71814, Cambridge, MA) and the Cas9 nuclease was further subcloned into the yeast pRS414 vector in our lab. *MDM12* and *BRE5* mutations were constructed using the wild-type Cas9 nuclease from Dr. George Church’s group [88]. CRISPR-Cas9-reconstituted clones were screened by PCR with either Donor F or R primer and an allele-specific primer that has the mutation site at the last position of the 3′ end and a mismatched nucleotide at the third last position to decrease the tendency of nonspecific annealing [91]. Fitness data were obtained by measuring at least three independent CRISPR-reconstituted clones.

## Supporting information

S1 Fig Expression of *Ylip-HSP90* increases hypersaline tolerance of *Saccharomyces cerevisiae* cells. (A) Growth rates of different replacement strains. *Ncas-HSP90* and *Klac-HSP90* replacement strains exhibited similar fitness to the *Scer-HSC82* strain under most conditions. In contrast, *Ylip-HSP90* replacement strains showed reduced fitness compared to the *Scer-HSC82* strain under most tested conditions but similar fitness in media containing 150 mM MgCl_2_, 1.8 M sorbitol, or 0.5 M NaCl. Unless specified otherwise, cells were grown at 28°C. Cells were grown in liquid cultures and growth rates were measured by plate readers. *p*-values were calculated by two-tailed Student *t* test with the Benjamini-Hochberg correction. *p < 0.05. **p < 0.01. ***p < 0.001. Data sets did not deviate from the normal distribution (Shapiro-Wilk test). Error bars are standard errors, *N* ≥ 4. The numerical data used in the figure are included in S1 Data. See also S1 Table. *Klac*, *Kluyveromyces lactis*; *Ncas*, *Naumovozyma castellii*; *Scer*, *Saccharomyces cerevisiae*; *Ylip*, *Yarrowia lipolytica*.
(EPS)

S2 Fig The phenotypic differences between Ylip-Hsp90– and Scer-Hsc82–carrying cells are not due to differences in the expression level or activity of these two orthologs. (A) The protein levels of Scer-Hsc82 and Ylip-Hsp90 are not significantly different between the ancestral *Scer-HSC82* and *Ylip-HSP90* strains (*p* = 0.071, *z* = −1.807, two-tailed Wilcoxon rank-sum test). Total cell protein was extracted and examined by western blot. The Hsp90 protein was N-terminally fused with a TAP tag and detected by the anti-TAP antibody. G6PDH was used as the internal control. The TAP-Hsp90/G6PDH ratios were normalized to that of the *Scer-HSC82* strain, and the bottom panel shows quantitative data of the western blot. Error bar represents the standard error, *N* ≥ 3. (B) Scer-Hsc82 and Ylip-Hsp90 exhibit similar v-src folding activities (*p* = 0.827, *z* = −0.218, two-tailed Wilcoxon rank-sum test). Log-phase cells were grown in galactose-containing medium for 6 hours to induce the expression of v-src. The same amount of total cell protein was loaded for each sample and examined by western blot. The phospho-tyrosine and v-src were detected by anti-phospho-tyrosine and anti-v-src antibodies, respectively. The phospho-tyrosine/v-src ratios were normalized to that of the *Scer-HSC82* strain, and the right panel shows quantitative data of the western blot. Error bar represents the standard error, *N* = 3. (C) *Yarrowia lipolytica* cells have higher fitness than *Saccharomyces cerevisiae* cells in hypersaline conditions. Serially diluted cell cultures were spotted onto plates containing 1 M NaCl or 0.2 M LiCl and incubated at 25°C until colonies became visible. The numerical data used in panels (A, B) are included in S1 Data. G6PDH, glucose-6-phosphate dehydrogenase; Scer, *Saccharomyces cerevisiae*; TAP, Tandem affinity purification; Ylip, *Yarrowia lipolytica*.
(EPS)

S3 Fig The frequency dynamics of each evolved line. Two isogenic subpopulations labeled with red or green fluorescence protein were mixed in an approximately 1:1 ratio in the initial cultures and allowed to evolve in YPD at 28°C. The subpopulation that obtained a beneficial mutation would start expanding its frequency in the evolved culture. The proportions of subpopulations were monitored using flow cytometry. The y-axis shows the green subpopulation frequency and the x-axis shows the numbers of evolved generations. The control line con6 and evolving lines evo1, evo2, and evo12 were excluded because of culture contamination during evolution. The numerical data used in the figure are included in S1 Data. YPD, Yeast extract-Peptone-Dextrose.
(EPS)

S4 Fig The ploidy and *Ylip-HSP90* protein expression levels of evolved clones. (A) Evolved *Scer-HSC82* clones all became diploid or triploid, but most of the evolved *Ylip-HSP90* clones remained haploid. All ancestral strains were confirmed to be haploid by flow cytometry. (B) Diploid *Ylip-HSP90*–carrying cells exhibit lower fitness than haploid *Ylip-HSP90*–carrying cells (*p* = 0.003, *z* = 2.744, one-tailed Wilcoxon rank-sum test), while diploid and haploid *Scer-HSC82*–carrying cells show similar fitness. Cells were grown in liquid cultures, and growth rates were measured by plate readers. Error bar represents the standard error, *N* ≥ 4. (C) The protein level of Ylip-Hsp90 in the evolved clones is not significantly different from that of the ancestral strains (two-tailed Wilcoxon rank-sum test). G and R indicate the ancestral strains carrying the green and red fluorescent proteins, respectively. Total cell protein was extracted and examined by western blot. The Hsp90 protein was N-terminally fused with a TAP tag and detected by the anti-TAP antibody. G6PDH was used as the internal control. The TAP-Hsp90/G6PDH ratios were all normalized to that of the ancestral *Ylip-HSP90* strains, and the bottom panel shows quantitative data of the western blot. Error bar represents the standard error, *N* ≥ 3. The numerical data used in the figure are included in S1 Data. G6PDH, glucose-6-phosphate dehydrogenase; *Scer*, *Saccharomyces cerevisiae*; TAP, Tandem affinity purification; *Ylip*, *Yarrowia lipolytica*.
(EPS)

S5 Fig Representative microscope images of cells containing Hsp104-BFP foci and measurement of cell morphology. (A) After cells were treated at 37°C for 3 hours, images of Hsp104-BFP foci were acquired and analyzed using a custom-built module under the MetaXpress High-Content Image Acquisition and Analysis Software. (B) Evolved *Ylip-HSP90* clones show different levels of protein homeostasis restoration. Cells carrying Hsp104-BFP were grown at 25°C and then shifted to 37°C to induce heat adaptation. The fraction of cells containing Hsp104-BFP foci was counted at 0, 1, 2, and 3 hours after the temperature shift. The same data used in Fig 3C were used to plot this figure. Error bar represents the standard error, *N* ≥ 7. (C) After labeling the cell wall with the fluorescent dye (green circles), the ratio of the long versus short axes (yellow lines) of yeast cells was calculated by Calmorph. The numerical data used in panel (B) are included in S1 Data. BFP, blue fluorescent protein; *Ylip*, *Yarrowia lipolytica*.
(EPS)

S6 Fig The evolved phenotypes of *Ylip-HSP90* clones are more divergent than those of *Scer-HSC82* clones. (A) PCA analysis of the fitness values shows that all *Ylip-HSP90* clones evolved diverged phenotypes, scattered across the four principal component dimensions. Explanatory power is shown in brackets next to each principal component. (B) Fitness improvements of the evolved *Scer-HSC82* clones under 11 different growth conditions. Cells were grown in liquid cultures and growth rates were measured by plate readers. Error bars are standard errors, *N* ≥ 2. Drug abbreviations are the same as Fig 4A. Fitness improvement was calculated by comparing the fitness of the evolved clone to that of the ancestral *Scer-HSC82* strain. (C) Evolved *Ylip-HSP90* clones responded to various stress conditions more differently than evolved *Scer-HSC82* clones. The variance of fitness improvements of evolved *Scer-HSC82* or *Ylip-HSP90* clones in each condition is plotted. The evolved *Ylip-HSP90* clones have significantly greater variances of fitness improvements compared with evolved *Scer-HSC82* clones (*p* = 0.039, one-tailed Student *t* test). (D) Evolved *Ylip-HSP90* clones have greater Pearson correlation distances between different conditions than evolved *Scer-HSC82* clones. The result suggests that evolved *Ylip-HSP90* clones generally have different fitness distribution patterns under different conditions, whereas the fitness patterns of evolved *Scer-HSC82* clones are similar to each other regardless of the stress types. Pearson correlation distances in all possible pairs of conditions were pooled and compared between evolved *Ylip-HSP90* and *Scer-HSC82* clones (*p* = 0.001, one-tailed Wilcoxon rank-sum test). The numerical data used in the figure are included in S1 Data. PC, principal component; PCA, principal component analysis; *Scer*, *Saccharomyces cerevisiae*; *Ylip*, *Yarrowia lipolytica*.
(EPS)

S7 Fig Summary of mutation types and numbers in evolved *Ylip-HSP90* clones. The genomes of evolved clones were sequenced using Illumina sequencers, and mutations were identified. The numerical data used in the figure are included in S1 Data. N, nonsynonymous mutation; S, synonymous mutation; *Ylip*, *Yarrowia lipolytica*.
(EPS)

S8 Fig Individual mutations have strong effects in different stress condition. The fitness improvement data of Fig 6A were used to draw this figure. The height of the bar represents the level of fitness improvements in a specific condition. Blue and red colors indicate the positive and negative effects, respectively. Error bars are standard errors, *N* ≥ 3. The numerical data used in the figure are included in S1 Data.
(EPS)

S1 Table Fitness measurements of cells carrying different Hsp90 orthologs under 17 growth conditions. Hsp90, heat shock protein 90.
(XLSX)

S2 Table Fitness measurements of evolved clones under 11 growth conditions. (XLSX)

S3 Table Correlation tests between the protein abundance of Ylip-Hsp90 and the fitness of evolved *Ylip-HSP90* clones in individual conditions. Ylip, *Yarrowia lipolytica*.
(XLSX)

S4 Table Summary of PCA analysis of the evolved clones. PCA, principal component analysis.
(XLSX)

S5 Table Pearson correlation distances and *p*-values. (XLSX)

S6 Table Mutation list of evolved *Ylip-HSP90* clones. *Ylip*, *Yarrowia lipolytica*.
(XLSX)

S7 Table Copy number variation in evolved clones. (XLSX)

S8 Table GO enrichment analysis of the protein-sequence-changing evolved mutations. GO, gene ontology.
(XLSX)

S9 Table Fitness measurements of the F1 segregants. (XLSX)

S10 Table Allele frequency thresholds from computational simulations and allele frequencies in the F1 segregant pools. (XLSX)

S11 Table Fitness measurements of reconstituted mutant strains under 11 growth conditions. (XLSX)

S12 Table Strain, guide RNA, and primer lists. (XLSX)

S1 Data Excel spreadsheet containing, in separate sheets, the underlying numerical data of Figs 1B, 2B, 2C, 3B, 3C, 3D, 4A, 4B, 4C, 4D, 6A, 6B, 6C, S1, S3, S5B, S6A, S6D, S8, S2A, S2B, S4A, S4B, S4C, S6B, S6C and S7. (XLSX)

## Abbreviations

AZC: azetidine-2-carboxylate
BFA: brefeldin A
BFP: blue fluorescent protein
Cas9: CRISPR-associated protein 9
CRISPR: clustered regularly interspaced short palindromic repeats
CSM: complete supplement mixture
GFP: green fluorescent protein
GO: gene ontology
HGT: horizontal gene transfer
Hsp90: heat shock protein 90
Klac: *Kluyveromyces lactis*
Ncas: *Naumovozyma castellii*
OD: optical density
PCA: principal component analysis
Scer: *Saccharomyces cerevisiae*
TetO_7_: tetracycline operator 7 repeats
Ylip: *Yarrowia lipolytica*
YPD: Yeast extract-Peptone-Dextrose
